# Combined Neuropeptide S and D-Cycloserine Augmentation Prevents the Return of Fear in Extinction-Impaired Rodents: Advantage of Dual versus Single Drug Approaches

**DOI:** 10.1093/ijnp/pyv128

**Published:** 2015-12-01

**Authors:** Simone B. Sartori, Verena Maurer, Conor Murphy, Claudia Schmuckermair, Patrick Muigg, Inga D. Neumann, Nigel Whittle, Nicolas Singewald

**Affiliations:** Department of Pharmacology and Toxicology, Institute of Pharmacy and Center for Molecular Biosciences Innsbruck (CMBI), Leopold-Franzens-University of Innsbruck, Innsbruck, Austria (Dr Sartori, Ms Maurer, Mr Murphy, and Drs Schmuckermair, Muigg, Whittle, and Singewald); Department of Behavioral and Molecular Neurobiology, University of Regensburg, Regensburg, Germany (Dr Neumann).

**Keywords:** Fear extinction, fear relapse, D-cycloserine, neuropeptide S, renewal

## Abstract

**Background:**

Despite its success in treating specific anxiety disorders, the effect of exposure therapy is limited by problems with tolerability, treatment resistance, and fear relapse after initial response. The identification of novel drug targets facilitating fear extinction in clinically relevant animal models may guide improved treatment strategies for these disorders in terms of efficacy, acceleration of fear extinction, and return of fear.

**Methods:**

The extinction-facilitating potential of neuropeptide S, D-cycloserine, and a benzodiazepine was investigated in extinction-impaired high anxiety HAB rats and 129S1/SvImJ mice using a classical cued fear conditioning paradigm followed by extinction training and several extinction test sessions to study fear relapse.

**Results:**

Administration of D-cycloserine improved fear extinction in extinction-limited, but not in extinction-deficient, rodents compared with controls. Preextinction neuropeptide S caused attenuated fear responses in extinction-deficient 129S1/SvImJ mice at extinction training onset and further reduced freezing during this session. While the positive effects of either D-cycloserine or neuropeptide S were not persistent in 129S1/SvImJ mice after 10 days, the combination of preextinction neuropeptide S with postextinction D-cycloserine rendered the extinction memory persistent and context independent up to 5 weeks after extinction training. This dual pharmacological adjunct to extinction learning also protected against fear reinstatement in 129S1/SvImJ mice.

**Conclusions:**

By using the potentially nonsedative anxiolytic neuropeptide S and the cognitive enhancer D-cycloserine to facilitate deficient fear extinction, we provide here the first evidence of a purported efficacy of a dual over a single drug approach. This approach may render exposure sessions less aversive and more efficacious for patients, leading to enhanced protection from fear relapse in the long term.

## Introduction

Anxiety disorders are the most common class of mental disorders in industrialized societies (Kessler et al., 2005; Wittchen et al., 2011). Diverse interventions are available for their treatment with significant advances in the last decades (Ravindran and Stein, 2010). Even though pharmacotherapy with benzodiazepines, monoamine oxidase inhibitors, selective serotonin and/or noradrenaline reuptake inhibitors, and tricyclics has demonstrated efficacy (Bandelow et al., 2012), exposure therapy is a particularly efficacious intervention to treat anxiety- and trauma-related disorders such as posttraumatic stress disorder, specific phobias, and social anxiety disorder (Olatunji et al., 2010; Abramowitz, 2013; Cuijpers et al., 2013). Still, in the everyday practice, exposure therapy struggles with limited acceptance by patients as well as psychologists due to its demanding and in some cases exhausting nature. Furthermore, only partial response and relapse in the long term have been reported (Choy et al., 2007; Stein et al., 2009; Bandelow et al., 2012).

The main process underlying exposure therapy is extinction. During an exposure session, the patient is repetitively confronted with the feared stimulus (eg, conditioned stimulus [CS]) in the absence of the harmful stimulus (unconditioned stimulus [US]). Thereby, the individual learns that the CS no longer predicts danger and builds a novel safety-based “CS-no US” memory that suppresses the original fear-eliciting CS-US association. Consequently, fearful responses decrease during an exposure session and between sessions, which is indicative of a successful exposure therapy (Abramowitz, 2013). In anxiety patients, these extinction mechanisms are often impaired, which manifests as a partial deficit or even lack in extinction learning and/or the consolidation of an extinction memory (Milad et al., 2013, reviewed in Holmes and Singewald, 2013). Furthermore, the original CS-US memory can predominate again, and fear returns after changes in the extinction context (fear renewal), the passage of time (spontaneous recovery), and stressful experiences (reinstatement) (Vervliet et al., 2013).

In an attempt to improve the treatment efficacy of exposure therapy, it seems to be practical combining exposure therapy with pharmacotherapy (for recent review, see Singewald et al., 2015). For example, it was hoped to counterbalance the aversiveness of the feared situation by acute anxiolytic effects of benzodiazepines given either prior to or during the exposure (Farrell et al., 2013), thereby increasing the patients’ compliance. However, the combination of exposure therapy with benzodiazepines (but also with many antidepressants) has yielded disappointing effects, as these drugs can interfere with the extinction-related mechanisms and thus degrade the long-term outcome of exposure therapy (Hofmann et al., 2009). As a result of intense animal research, the FDA-approved D-cycloserine (DCS), a partial N-methyl-D-aspartate receptor agonist, has shown promise in augmenting exposure therapy of anxiety disorders, including specific phobias and social anxiety disorder, by strengthening the crucial learning processes underlying extinction (Davis et al., 2006; Hofmann et al., 2013; Singewald et al., 2015). Despite the obvious success story for translational science, failures of DCS are also reported in anxiety patients and open questions remain in terms of its long-term effects, dose, timing, and interaction with exposure sessions (Hofmann et al., 2013; de Kleine et al., 2015; MacKillop et al., 2015).

Apart from DCS, there are several other drug targets of interest whose potential in promoting fear extinction is currently studied in animals, including monoamines, cannabinoids, steroids, neurotrophins, and neuropeptides (Singewald et al., 2015). Among the latter, neuropeptide S (NPS) represents an interesting substance, as it induces anxiolysis and arousal (Reinscheid et al., 2005; Adori et al., 2015) and facilitates fear extinction learning and retrieval in mice when applied into the amygdala (Jungling et al., 2008; Chauveau et al., 2012). Moreover, polymorphisms in the NPS receptor gene leading to altered receptor function are suggested to contribute to the pathological mechanisms involved in anxiety disorders, including panic disorder and posttraumatic stress disorder (Okamura et al., 2007; Donner et al., 2010; Raczka et al., 2010; Dannlowski et al., 2011; Domschke et al., 2011; Glotzbach-Schoon et al., 2013; Slattery et al., 2015).

Here, we aimed at further exploring the potential of DCS and NPS in augmenting fear extinction. For this purpose, we used 2 different rodent models with individual differences in extinction efficacy and subjected them to fear extinction (following Pavlovian fear conditioning) and extinction retrieval sessions. The rodent models of impaired fear extinction were (1) rats selectively bred for high anxiety-related behavior (HAB), which show some, though considerably decelerated, fear extinction learning compared with their low-anxiety (LAB) controls (Muigg et al., 2008); and (2) the 129S1/SvImJ (S1) mouse, which shows deficient fear extinction acquisition and/or impaired extinction consolidation modelling treatment resistance depending on the conditioning paradigm applied (Hefner et al., 2008; Camp et al., 2009; Whittle et al., 2010, 2013). Stimulated by the results of the first set of data of the current study demonstrating short-term benefit of NPS or DCS on fear extinction in both animal models, we next hypothesized that DCS may strengthen consolidation processes triggered by the anxiolytic and extinction-inducing effects of NPS. Thereto, we decided to pursue subsequent long-term effects of NPS and DCS in the severely extinction-deficient S1 mouse.

## Methods

### Animals

Adult male HAB and LAB rats (University of Regensburg, Regensburg, Germany) with confirmed anxiety phenotype as assessed on the elevated plus maze (for further details, see Neumann et al., 2010) and adult male S1 mice (University of Innsbruck, Innsbruck, Austria) were used in these studies. Animals were group-housed under standard laboratory conditions (12:12 light/dark cycle with lights on at 7:00 am, 22±2°C, 50–60% humidity) and had free access to food and water. All experiments were designed to minimize animal suffering as well as the number of animals used and were approved by the Austrian national ethical committee on animal care and use (Bundesministerium für Wissenschaft und Forschung) in compliance with international laws and policies.

### Auditory Fear Conditioning

The auditory fear conditioning experiments were carried out according to previous protocols for rats (Muigg et al., 2008) and mice (Whittle et al., 2013). In all sessions, 120-second stimulus-free habituation periods and consolidation periods were allowed prior to and after the last stimulus presentations. In HAB and LAB rat experiments, animals received 5 auditory cues (CS; white noise, 80 dB, 30 seconds) that each coterminated with a mild, short, scrambled foot shock (US; 0.7 mA, 2s) in water-cleaned conditioning chambers (26×30×32cm; Coulbourn Instruments, Allentown, PA) under bright illumination (300 lux). Twenty-four hours later (day 2), extinction training of cued conditioned fear was performed in standard rat cages (22×37.5×15cm; wiped with ethanol; illuminated with red light to 10 lux), where animals were exposed to 30 CSs in the absence of the US each separated by an inter-trial interval of 5 seconds. On day 3 of the experiment, 2 CSs were presented to animals in the extinction context to test for extinction retrieval. The presentation of stimuli was controlled by the Habitest operant system (Coulbourn Instruments, Allentown, USA).

**Figure 1.**
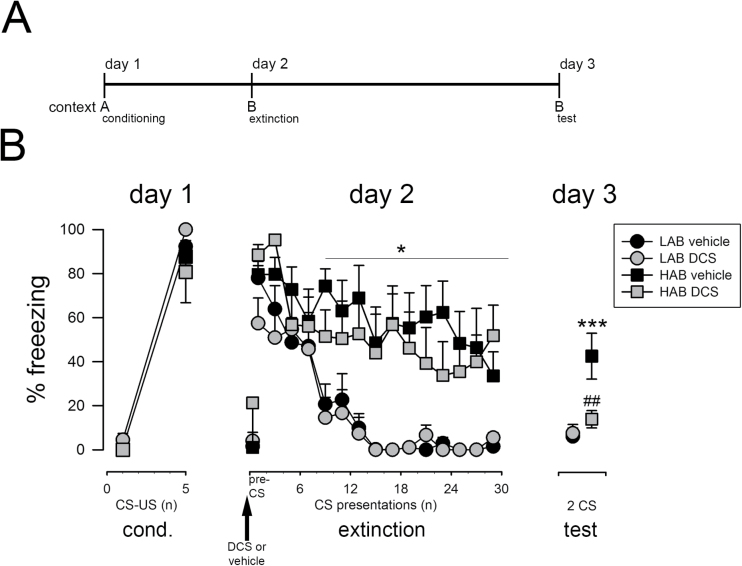
Effect of D-cycloserine (DCS) on fear extinction in high-anxiety (HAB) and low-anxiety (LAB) rats. (A) Schematic representation of the experimental design. (B) HAB and LAB rats acquired conditioned fear upon 5 conditioned stimulus (CS)-unconditioned stimulus (US) pairings. Although the systemic application of DCS (15mg/kg; ip) before extinction training did not affect the extinction training per se, it reduced freezing to the CS in HAB rats on test day 3, pointing towards facilitated fear extinction. Data are means ± SEM, n = 6 to 7/experimental group. *P<.05 and ***P<.001 for HAB vs LAB groups, ^##^ *P*<.01 for drug treatment vs vehicle treatment (multiple-factor ANOVA with repeated measures and post-Fisher’s LSD test). A: context A; B: context B; cond, conditioning.

**Figure 2.**
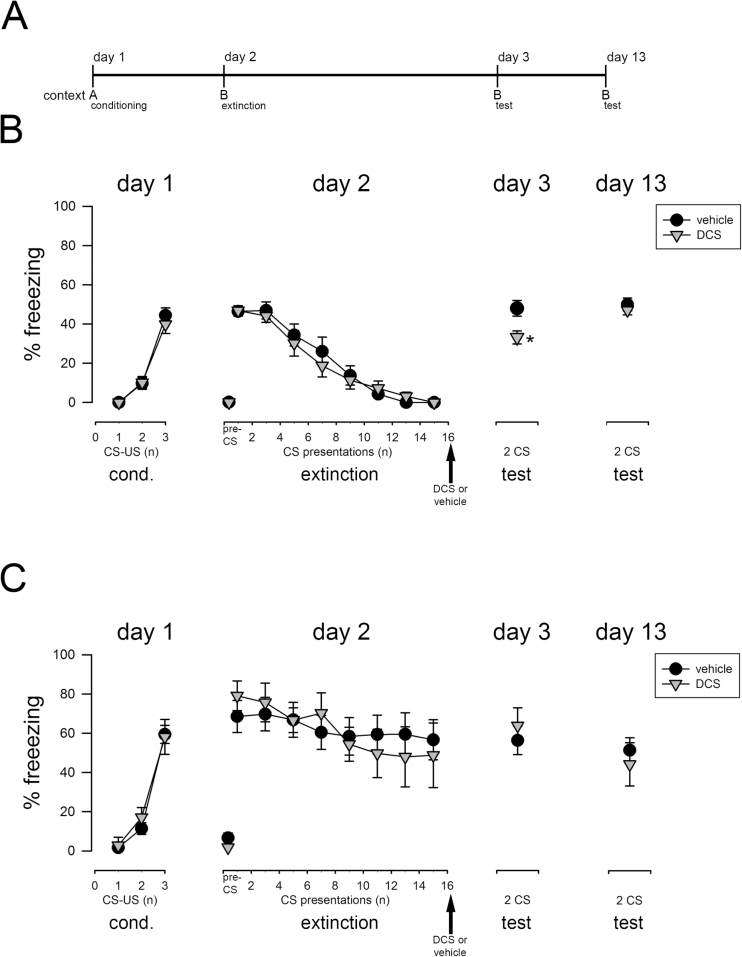
Effect of D-cycloserine (DCS) on fear extinction in extinction-impaired 129S1/SvImJ (S1) mice. (A) Schematic representation of the experimental design. (B) In extinguishing S1 mice (following weak fear conditioning), the postextinction application of DCS (15mg/kg; ip) caused decreased freezing to the conditioned stimulus (CS) on test day 3, indicating facilitated fear extinction. However, the CS-induced fear response returned in DCS-treated S1 mice on test day 13. (C) The systemic application of DCS (30mg/kg; ip) immediately postextinction training did not affect the freezing to the CS on test days 3 and 13 in extinction-deficient S1 mice (following normal fear conditioning). Data are means ± SEM, n = 6 to 11/experimental group. *P<.05 for DCS treatment vs vehicle treatment. A: context A; B: context B; cond: conditioning; US: unconditioned stimulus.

**Figure 3.**
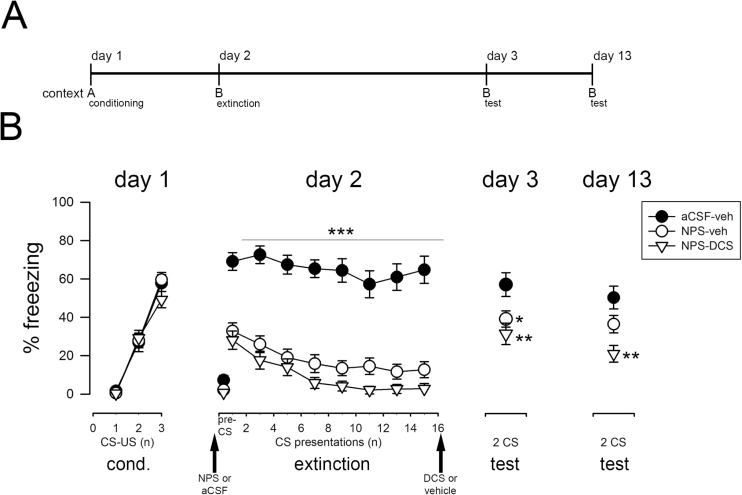
Effects of combined D-cycloserine (DCS) and neuropeptide S (NPS) on fear extinction in extinction-deficient 129S1/SvImJ (S1) mice. (A) Schematic representation of the experimental design. (B) The intra-cerebral infusion of NPS (1 nmol; intra-cerebroventricular) before extinction training caused a pronounced decrease in freezing at the beginning of the extinction training session and a further decline within the session, indicating acute anxiolysis followed by fear extinction. Freezing to the conditioned stimulus (CS) was reduced in NPS-treated S1 mice on test day 3, but not on test day 13. In contrast, the systemic application of DCS (30mg/kg; ip) immediately after NPS-induced fear extinction caused a significant reduction in freezing displayed by S1 mice on both test days. Data are means ± SEM, n = 14 to 16/experimental group. *P<.05, **P<.01, and ***P<.001 for NPS-vehicle or NPS-DCS treatment vs artificial cerebrospinal fluid (aCSF)-vehicle controls. A: context A; B: context B; cond: conditioning; DCS: D-cycloserine; ip: intra-peritoneal; NPS: neuropeptide S; US: unconditioned stimulus; veh: vehicle.

**Figure 4.**
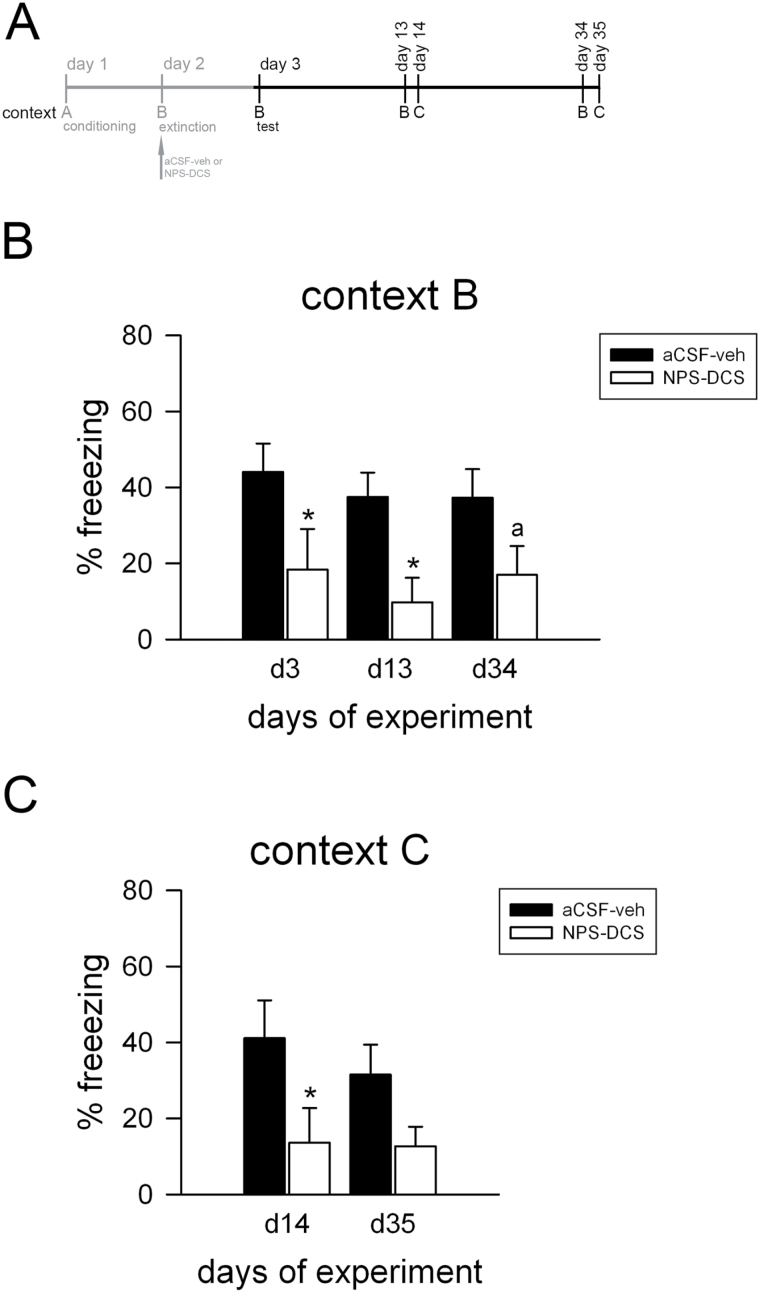
D-cycloserine (DCS) adjunction to neuropeptide S (NPS) produced long-term fear extinction and generalization of extinction in 129S1/SvImJ (S1) mice. (A) Schematic representation of the experimental design. The systemic application of DCS (30mg/kg; ip) immediately after NPS- (1 nmol; intra-cerebroventricular prior to fear extinction training) induced fear extinction caused reduced freezing in the extinction context B (B) as well as in a novel context C (C), indicating generalization of fear extinction promoting in the long-term in S1 mice. Data are means ± SEM, n = 8 to 9/experimental group. ^a^ *P*<.08 and *P<.05 for NPS-DCS treatment vs artificial cerebrospinal fluid (aCSF)-vehicle controls. A: context A; B: context B; C: context C; veh: vehicle.

**Figure 5.**
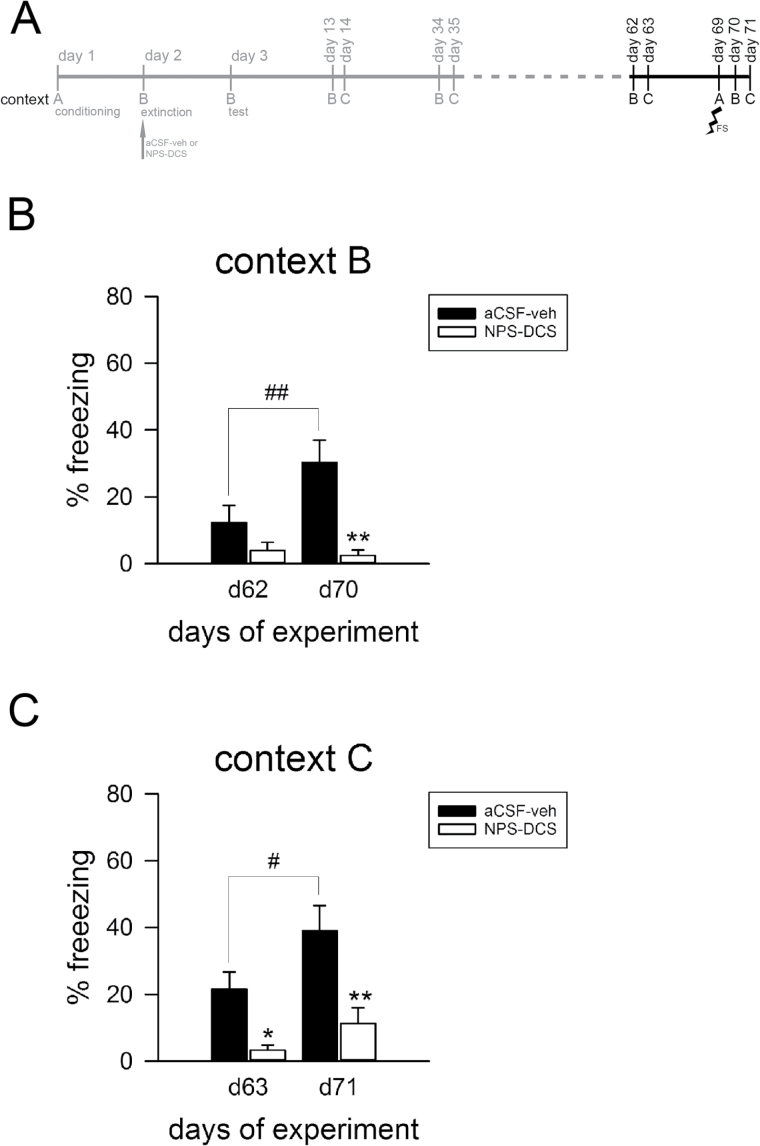
D-cycloserine (DCS) adjunction to neuropeptide S (NPS)-induced fear extinction prevented the reinstatement of cued conditioned fear in 129S1/SvImJ (S1) mice. (A) Schematic representation of the experimental design. The data of those parts plotted in grey are not shown here but in Figure 4. (B-C) Following a single unsignaled unconditioned stimulus (US) in the conditioning context A, control (artificial cerebrospinal fluid [aCSF]-veh) mice displayed increased freezing in context B (B) and context C (C) in response to the CS, indicating reinstatement of cued conditioned fear. In contrast, the systemic application of DCS (30mg/kg; ip) immediately after NPS- (1 nmol; intra-cerebroventricular prior extinction training) induced fear extinction caused low levels of freezing in both contexts B and C following a single unsignaled US in the conditioning context A, indicating protection from return of fear. Data are means ± SEM, n = 6 to 9/experimental group. *P<.05 and **P<.01 for NPS-DCS treatment vs aCSF-vehicle controls, ^#^ *P*<.05 and ^##^ *P*<.01 for pre- vs postreinstatement of the same experimental group. A: context A; B: context B; C: context C; d: day; FS: (unsignaled) footshock; veh: vehicle.

Mouse fear conditioning experiments were performed in fear conditioning systems (TSE Systems GmbH, Bad Homburg, Germany). Mice acquired fear following 3 pairings of a tone CS (10kHz, sine tone; 65 dB, 30 seconds) and a mild footshock US (0.6 mA for standard conditioning and 0.3 mA for weak fear conditioning, pulsed, 2 seconds; Whittle et al., 2013) with a 120-second inter-trial interval in context A (25×25×35cm chamber with transparent walls and a metal rod floor, cleaned with water and illuminated to 300 lux). On the next day (day 2), 16 CSs alone separated by 5 seconds were presented to mice for a fear extinction test in a novel context B (25×25×35cm chamber with black walls and a solid grey floor, cleaned with 100 % ethanol and illuminated to 10 lux). An extinction retrieval test was performed on day 3 by presenting 2 CSs separated by 5 seconds in context B. In the following weeks, long-term effects on extinction retrieval, generalization of extinction, and fear reinstatement were tested by presenting 2 CSs either in context B on experimental days 13, 34, 62, and 70 or in a novel context C (35×20cm round Plexiglass cylinder with pale-colored tiles on the floor and red diamond checks on the walls) on experimental days 14, 35, 63, and 71. For fear reinstatement, an unsignaled US (0.6 mA, pulsed, 2 seconds) was presented to the animals in context A. The chronological design of each experiment is presented in the figures (Figures 1A, 2A, 3A, 4A, and 5A; supplementary Figure 1A).

#### Behavioral Analysis

The behavior of animals was recorded via video cameras positioned above the experimental contexts. A trained observer blinded to treatment groups determined the time animals remained in freezing behavior, that is, no visible movements except those needed for respiration, as an index of fear (Blanchard and Blanchard, 1969), and it was converted into a percentage of each CS period. Freezing scores during extinction and extinction retrieval were binned into blocks, each representing the mean value of 2 consecutive CSs.

### Drug Treatments

DCS (15mg/kg for rats and 15 or 30mg/kg for mice, as both doses have been shown to be similarly effective; Singewald et al., 2015; dissolved in saline; Sigma) was injected i.p. 20 minutes before or immediately after the extinction training. NPS (1 nmol; Bachem) was dissolved in artificial cerebrospinal fluid (140mM NaCl, 3mM KCl, 1.25mM CaCl_2_, 1mM MgCl_2_, 1.2mM Na_2_PO_4_, 0.3mM NaH_2_PO_4_, 3mM glucose, pH 7.2) and infused (2 μL at a rate of 1 μL/min) into the lateral ventricle of mice 20 minutes prior to extinction training via micro-cannulae (30 G, 12mm long) whose tip extended past the guide cannulae by 1mm and which was connected to Hamilton syringes. Control animals received vehicle via the respective application route.

### Surgery

For intra-cerebral infusion of NPS, indwelling guide cannulae (23 G, 8mm long) were implanted above the right lateral ventricle (coordinates: -0.8mm caudal, 1.5mm lateral, 1.2mm ventral from bregma according to Paxinos and Franklin, 2001) in anaesthetized (5mg/kg xylazine, 80mg/kg ketamine, i.p.) S1 mice. Animals received buprenorphine (0.5mg/kg every 8 hours for 3 days; i.p.) for postoperative analgesic care. They were allowed to recover for at least 5 days until testing and were daily handled to familiarize them to the experimental procedure. At the end of the behavioral experiments, blue dye was injected via the microinfusion system for verification of the infusion sites. Only animals with correct placement of the microcannulae (95% of animals) into the lateral ventricle were considered for behavioral analysis.

### Statistical Analysis

The percentage of freezing is presented as mean ± SEM. Pre-CS freezing as well as the freezing on test days 3 and 13 were analyzed using either ANOVA for multiple group-comparisons or an independent-samples 2-tailed *t* test for 2 group comparisons. Differences in fear acquisition, extinction training, extinction retrieval, and fear reinstatement were analyzed using a repeated-measures ANOVA. When applicable, posthoc comparisons in ANOVA were performed using the LSD test. Level of statistical significance was set to *P* < .05. Degrees of freedom may vary within an experiment, because the behavior of some animals could not be analyzed in each test session.

## Results

### Effect of DCS on Fear Extinction in HAB and LAB Rats

In the first set of experiments, we examined the effect of DCS on the extinction of cued conditioned fear (Figure 1A) in HAB rats with high trait anxiety and impaired extinction learning and used the low-anxiety counterparts (LAB rats) as comparison. Freezing levels increased upon 5 CS-US pairings (pairing effect: F_4,88_= 72.3, *P*<.001) and reached similar high levels in HABs and LABs (line × pairing effect: F_4,88_ = 0.804, *P*>.05), indicating successful fear acquisition in both lines (Figure 1B). In line with our previous studies (Muigg et al., 2008; Slattery et al., 2015), the repeated presentation of the CS in the absence of the US for extinction training induced a fast decline in freezing of LAB rats, while elevated freezing levels persisted in HABs (Figure 1B). This difference in fear expression was also evident on test day 3 (Figure 1B). Preextinction administration of DCS did not affect pre-CS freezing to the context B (line x treatment interaction: F_1,23_ = 4.17, *P*>.05; Figure 1B) and did not alter the characteristic changes in CS-provoked freezing of HABs and LABs during extinction training as analyzed by multiple-factor ANVOA with repeated measures (CS × treatment interaction: F_14,322_ = 0.429, *P*>.05; Figure 1B). However, at extinction retrieval on test day 3, DCS-treated HAB rats displayed lower freezing than vehicle-treated HABs and similar freezing levels to LABs (line × treatment interaction: F_1,22_ = 5.32, *P*=.031; Figure 1B), suggesting an extinction memory promoting effect of DCS in extinction-impaired HAB rats.

### Effect of DCS on Fear Extinction Consolidation in Extinction-Impaired S1 Mice

We next investigated the effect of DCS in S1 mice that either fail to consolidate a new extinction memory (Whittle et al., 2013) or do not show any extinction learning at all (Hefner et al., 2008; Camp et al., 2009) depending on the conditioning paradigm (Figure 2A). This time, however, DCS was applied after the extinction training, since in our previous study we demonstrated that DCS before extinction training was ineffective in facilitating fear extinction in S1 mice (Hefner et al., 2008). S1 mice acquired cued conditioned fear within 3 pairings of a CS and a weak (0.3 mA) US (pairing effect: F_2,32_ = 121, *P*<.001; Figure 2B) as well as within 3 pairings of a CS and a standard (0.6 mA) US (pairing effect: F_2,32_ = 137, *P*<.001; Figure 2C). In extinction training, 24 hours after weak fear conditioning, S1 mice extinguished cued fear upon the repeated presentation of the unpaired CS in context B (CS effect: F_7,112_ = 55.9, *P*<.001; Figure 2B). In these extinguishing S1 mice, the postextinction DCS treatment caused lower freezing in extinction retrieval on experimental day 3 compared with vehicle-treated controls (t_16_ = 2.84, *P*=.012; Figure 2B). Ten days later, fear responses were increased in DCS-treated S1 mice reaching fear levels of controls (t_16_ = 0.725, *P*>.05; Figure 2B). This finding indicates that DCS rescued the impaired consolidation of extinction memory, but did not induce long-term extinction retrieval in S1 mice. In contrast, in S1 mice subjected to the standard cued fear conditioning protocol, the repeated presentation of the CS-only during extinction training did not affect freezing levels between the first and last CS block, and freezing levels also did not differ between treatment groups (CS × treatment interaction: F_7,112_ = 1.33, *P*>.05; Figure 2C), indicating no extinction of cued conditioned fear in S1 mice. Administration of DCS immediately after extinction training did not alter fear responses during the test sessions performed on experimental day 3 (t_16_ = -0.618, *P*>.05) and day 13 (t_11_ = 0.593, *P*>.05) (Figure 2C). These data confirm observations in our previous study in S1 mice (Hefner et al., 2008) suggesting that DCS does not exert its extinction-promoting effects in the absence of extinction learning.

### Effect of DCS and NPS on Fear Extinction in Extinction-Deficient S1 Mice

Stimulated by studies showing that intra-amygdaloid NPS facilitates fear extinction learning and retrieval in mice (Jungling et al., 2008; Chauveau et al., 2012), we tested whether NPS can exert beneficial effects also in the extinction-deficient S1 mice (Figure 3A) reflecting anxiety patients that are resistant to exposure therapy and thus that group of anxiety patients that is the most difficult to treat. Animals of all experimental groups developed freezing to the CS paired with the standard US (0.6 mA) to a similar extent during the conditioning sessions (pairing effect: F_2,134_ = 203, *P*<.001; pairing × treatment interaction: F_4,134_ = 1.24, *P*>.05; Figure 3B). NPS applied intra-cerebroventricularly before the extinction training had a small but statistically significant effect on pre-CS freezing to context B (F_2,68_ = 3.15, *P*=.049). Intra-cerebral NPS caused a pronounced decrease in freezing to the CS at the beginning of the extinction session and a further decline in freezing within the extinction session (CS × treatment interaction: F_14,476_ = 1.89, *P*=.026; Figure 3B). NPS-treated S1 mice displayed lower fear responses than controls in the retrieval test session performed on the next day (treatment effect: F_2,64_ = 4.18, *P*=.020; Figure 3B), indicating extinction memory formation. However, 10 days later, freezing levels were similar between NPS-vehicle and artificial cerebrospinal fluid (aCSF)-vehicle-treated S1 mice (treatment effect: F_2,41_ = 6.84, *P*=.003; Figure 3B), indicating that the NPS effects were not enduring.

Since DCS was able to promote fear extinction consolidation in extinguishing S1 mice in the previous experiment (Figure 2B), we wondered whether postextinction administration of DCS would be able to boost the consolidation of the NPS-induced extinction learning (Figure 3B). Again, NPS reduced freezing within the extinction training. Indeed, freezing remained lower in animals receiving combined postextinction DCS on top of preextinction NPS than in the aCSF vehicle-treated group (treatment effect: F_2,64_ = 4.18, *P*=.020; Figure 3B) on test day 3 (retrieval) and (treatment effect: F_2,41_ = 6.84, *P*=.003; Figure 3B) day 13 (long-term retrieval) as well as than the NPS vehicle-treated group on test day 13 (Figure 3B).

### Effect of Combined NPS and DCS Adjunction on the Return of Fear in Extinction-Deficient S1 Mice

In an attempt to study the long-term effectiveness of NPS-DCS-augmented extinction on the reemergence of fear in S1 mice, we next investigated the combined treatment effect on long-term generalization of fear extinction (Goode and Maren, 2014) (Figure 4A). For this purpose, S1 mice were fear conditioned using the standard protocol and subjected to an extinction training session with or without NPS-DCS administration the next day. Similar to the preceding experiment (Figure 3B), experimental groups acquired cued conditioned fear to a similar extent (aCSF-vehicle: 72±3.66%; NPS-DCS: 64±4%; pairing effect: F_2,48_ = 95.8, *P*<.001; pairing × treatment interaction: F_2,48_ = 0.598, *P*>.05) and NPS-DCS adjunction again facilitated fear extinction in extinction-deficient S1 mice as indicated by reduced fear responses at the end of extinction training (CS × treatment interaction: F_7,168_ = 11.0, *P*<.001) compared with vehicle treatment. Then, CS-elicited fear responses of NPS-DCS-treated and vehicle-treated S1 mice were assessed in the extinction context B and a novel context C until experimental day 35 (Figure 4). In context B, levels of freezing did not change over time (F_2,30_ = 0.687, *P*>.05). They remained high in control animals and low in NPS-DCS-treated animals on experimental days 3, 13, and 34 (Figure 4B) and significantly differed between the 2 experimental groups (treatment effect: F_1,15_ = 9.35, *P*=.008). Similarly, when the CS was presented in context C, which had not previously been associated with either fear conditioning or fear extinction, vehicle-treated S1 displayed pronounced freezing on experimental days 14 and 35 while attenuated freezing levels were observed in NPS-DCS-treated S1 mice (treatment effect: F_1,15_ = 8.52, P = 0.011; time effect: F_1,15_ = 0.361, *P*>.05) (Figure 4C). These findings show that DCS treatment on top of NPS-induced extinction learning is beneficial for generalization of fear extinction in the long-term in S1 mice.

Since fear may return after successful extinction when the original or another aversive stimulus is experienced (fear reinstatement) (Goode and Maren, 2014), we next presented a single footshock reminder to the same animals (Figure 4) in the conditioning context A on day 69 and investigated the effect of NPS-DCS adjunction on CS-elicited fear responses (Figure 5A). Freezing was low in the extinction context B on experimental day 62 (Figure 5B) and in the novel context C on experimental day 63 (Figure 5C), the days just before the presentation of an unpaired US in context A. The US did not lead to freezing to either context B (aCSF-veh: 0.06±0.06%; NPS-DCS: 0%) or to context C (aCSF-veh: 0.02±0.02%; NPS-DCS: 0%) prior to the first CS presentation, but it significantly increased CS-induced freezing in context B (F_1,13_ = 5.14, *P*=.041; Figure 5B) and context C (F_1,13_ = 7.66, *P*=.016; Figure 5C), indicating successful fear reinstatement. Fear reinstatement, however, was observed only in vehicle-treated S1 mice in context B (treatment effect: F_1,13_ = 7.57, *P*=.017 and US × treatment interaction: F_1,13_ = 7.04, *P*=.020), while S1 mice with the dual treatment remained at lower levels. Likewise, in context C, CS-induced freezing was significantly enhanced in aCSF-veh controls, but not in NPS-DCS-treated animals (treatment effect: F_1,13_ = 10.6, *P*=.006; Figure 5C), although no US × treatment interaction (F_1,13_ = 1.07, *P*>.05) was revealed. These findings show that a NPS-DCS adjunct treatment to extinction training also protected against fear reinstatement in S1 mice.

## Discussion

In line with previous studies in rodents and humans (Hofmann et al., 2013; Singewald et al., 2015), the present set of experiments demonstrated that DCS facilitated short-term fear extinction in rodents showing a decline in fear responses during an extinction training session (HAB rats or weakly conditioned S1 mice), while DCS could not promote fear extinction in extinction-resistant S1 mice. Preextinction intra-cerebroventricular infusion of NPS caused attenuated fear responses in extinction-deficient S1 mice already at the beginning of the extinction training and further reduced freezing during the session. However, fear responses returned in animals treated with either DCS or NPS. Here, we show for the first time that a dual approach – postextinction DCS on top of preextinction NPS treatment – caused a long-term facilitatory effect on fear extinction that generalized to a novel context and prevented extinction-deficient S1 mice from exhibiting fear reinstatement.

We have previously shown that DCS applied prior to an extinction training session does not cause extinction learning in S1 mice (Hefner et al., 2008). Preextinction DCS also did not modulate the decline in CS-elicited freezing of HAB and LAB rats during the extinction session. However, in the present set of experiments, we observed that DCS, regardless of whether it was applied before or immediately after successful fear extinction, promoted extinction consolidation in HAB rats and weakly conditioned S1 mice as evidenced in the extinction retrieval test on the next day. These findings replicate our former study in S1 mice (Whittle et al., 2013) and further reinforce studies in “normally” extinguishing rodents (Weber et al., 2007; Bouton et al., 2008) as well as a recent retrospective study in patients with acrophobia (Smits et al., 2013) suggesting that the efficacy of DCS depends on the success of the extinction training session. Consequently, DCS should not be administered before an extinction session with unknown outcome, but after an extinction session when this was successful in declining stimulus-elicited fear responses (Hofmann et al., 2013).

In contrast to DCS, intra-cerebral infusion of NPS reduced CS-elicited freezing already at the beginning of the extinction session in extinction-deficient S1 mice. Central administration of NPS has been reported to enhance vigilance (Reinscheid et al., 2005) and produce anxiolytic effects in rodents in various paradigms, including the elevated plus maze, open field test, light-dark box, marble burying, and stress-induced hyperthermia (Jungling et al., 2008; Leonard et al., 2008; Vitale et al., 2008; Ruzza et al., 2010; Lukas and Neumann, 2012; Wegener et al., 2012; Slattery et al., 2015). Since many patients do not accept exposure therapy due to either the anticipated or actual distress associated with being confronted to the feared situation by the exposure (Farrell et al., 2013), the initial fear-reducing effect of NPS may have considerable clinical impact by potentially increasing the tolerability of exposure therapy. Indeed, there have been various attempts to combine exposure therapy with fast-acting anxiolytics such as diazepam in order to increase its tolerability. This approach, however, has revealed disappointing outcomes as benzodiazepines seem to interfere with extinction learning processes, probably involving state-dependent mechanisms (Goldman, 1977; Pereira et al., 1989; Bouton et al., 1990; Bustos et al., 2009; Hart et al., 2014). In line with previous observations in animals (Pereira et al., 1989; Bouton et al., 1990) and humans (Spiegel and Bruce, 1997; Birk, 2004), diazepam also impaired fear extinction in LAB rats and decelerated extinction acquisition in HAB rats (supplementary Figure 1), further supporting the value of the HAB/LAB model in translational research for investigating extinction-facilitating drugs. Reasons for these undesired effects of diazepam may be its sedative properties and/or state-dependency (Birk, 2004; Hofmann et al., 2013). NPS is probably the only potential fast-acting anxiolytic so far that has been shown to induce arousal rather than sedation (Reinscheid et al., 2005). Interestingly, when anxiety patients are in an emotionally excited state similar to that as during the formation of the initial fear memory, extinction learning is more effective (Foa and Kozak, 1986), raising the exciting idea that despite its fear-reducing properties, NPS also promotes the building of fear extinction memories. Likewise, there is evidence for similar actions of the fibroblast growth factor-2 (Graham and Richardson, 2009, 2010).

We now found that central NPS was able to facilitate fear extinction in the extinction-deficient S1 mouse, which was observed both during extinction acquisition and consolidation and was indicated by reduced freezing compared with vehicle-treated mice. Interestingly, NPS has been shown to accelerate extinction learning in naturally extinguishing rodents without affecting fear acquisition or the consolidation or recall of conditioned fear (Jungling et al., 2008; Slattery et al., 2015). Similarly, the impaired extinction acquisition and extinction consolidation of S1 mice can be rescued by preextinction administration of the GABAergic enhancer valproic acid, the α2-adrenoreceptor antagonist yohimbine, the metabotropic glutamate receptor 7 agonist AMN082 or the fatty acid amide hydrolase AM3506 (Hefner et al., 2008; Gunduz-Cinar et al., 2013; Whittle et al., 2013) as well as chronic treatment with the selective serotonin reuptake inhibitor fluoxetine (Camp et al., 2012). We thus suggest that NPS promoted the acquisition and maybe also the consolidation of the extinction memory rather than interfering with the initial CS-US memory. In support of this idea, promnestic properties of central NPS are reported in incidental and spatial learning (Okamura et al., 2011; Lukas and Neumann, 2012; Han et al., 2013). Alternatively, it may also be that NPS-treated mice now associated the extinction context with the activity-inducing effects of NPS (Reinscheid et al., 2005), as it was reported with yohimbine (Morris and Bouton, 2007). However, this idea is very unlikely, since NPS-DCS treated animals also showed reduced CS-elicited fear in the novel context C. Regarding the possible mechanisms underlying the reduced fear expression and the facilitated acquisition and consolidation of extinction in S1 mice, NPS enhances, most likely via stimulation of select intercalated GABAergic populations, the feed-forward inhibition of projection neurons of the central amygdala, which is the main output nucleus of the amygdala connecting with various forebrain and brain stem structures eliciting fear responses (Jungling et al., 2008; Meis et al., 2008). In addition, NPS has also been shown to engage some other critical substrates of the extinction neurocircuitry (Graham and Milad, 2011; Herry and Johansen, 2014), including central noradrenaline (Okamura et al., 2011) and dopamine neurotransmission in the medial prefrontal cortex (Si et al., 2010) or ventral hippocampus (Dine et al., 2015).

Nevertheless, the extinction-promoting effects of both DCS and NPS were relatively short-lived in extinction-deficient S1 mice, and fear responses were then no longer different from those of vehicle-treated animals, indicating that neither DCS nor NPS erased the initial CS-US association in S1 mice, which is expressed as freezing. Return of fear phenomena is also frequently observed following initially successful exposure therapy in patients (Vervliet et al., 2013). Since these drugs act on different pharmacological targets, we wondered whether NPS and DCS adjunction would exert synergistic effects in augmenting long-term fear extinction in extinction-deficient individuals. Indeed, relative to NPS alone or vehicle treatment, NPS-DCS-treated S1 mice showed good extinction-retrieval in the long-term, as their levels of freezing were reduced in the extinction context throughout the entire experimental time frame (more than a month) compared with vehicle-treated controls. Furthermore, extinguished fear did not increase with the change to a novel context, and following an unpaired presentation of the US in NPS-DCS-treated S1 mice compared with controls pointing towards generalization of fear extinction. These findings suggest that dual NPS-DCS adjunction to extinction training protected against different forms of fear relapse in the laboratory. Considering the promnestic properties of both NPS (Okamura et al., 2011; Lukas and Neumann, 2012; Han et al., 2013) and the partial N-methyl-D-aspartate receptor agonist DCS (Monahan et al., 1989; Flood et al., 1992; Quartermain et al., 1994; Pitkanen et al., 1995; Pussinen et al., 1997; Land and Riccio, 1999) in rats and mice, we suggest that the dual NPS-DCS adjunction to extinction training allowed the formation of a stronger CS-no US extinction memory than single NPS or DCS treatment. The neuronal mechanisms underlying this robust memory are not clear, but it may be speculated that DCS strengthened NPS-initiated memory-promoting mechanisms such as long-term potentiation in extinction-relevant brain areas, including the medial prefrontal cortex, amygdala, and hippocampus during fear extinction by simultaneously targeting diverse neurotransmitter systems such as monoamines, glutamate, and GABA (Jungling et al., 2008; Meis et al., 2008; Si et al., 2010; Okamura et al., 2011). Alternatively, it may well be that rather than reflecting interactive or even synergistic mechanisms of NPS-DCS adjunction, additive effects in terms of more treatment in general may be responsible for the strong extinction retrieval that needs to be addressed in subsequent studies. Specifically, it would be interesting to see whether a higher dose of either drug alone had an effect comparable with that produced by the combined drug treatment used here. This approach, however, may be problematic, as higher doses (as well as repeated administration) of DCS increase the risk of side effects and of tolerance and have been reported to exert weak, in part even smaller, effects than lower doses of DCS or even placebo (Hofmann et al., 2013).

Taken together, we provide the first evidence that a dual-drug approach using both preextinction NPS and postextinction DCS may be more beneficial than NPS or DCS as stand-alone treatments in augmenting fear extinction retrieval in extinction-impaired animals. Combining the therapeutic actions of a potentially nonsedative anxiolytic drug and cognitive enhancers is an absolutely novel concept in order to promote fear extinction and thus may have profound clinical impact for patients with anxiety disorders by means of increasing tolerability of exposure-based therapy and reducing fear relapse in the long- term. An important following step is the development of small, brain-penetrant, nonpeptidergic NPS receptor agonists, which ultimately may be applied to patients with anxiety disorders. Next, it is of great interest to elucidate the mechanisms underlying the synergistic effects of NPS-DCS adjunction to fear extinction compared with stand-alone pharmacotherapy.

## Statement of Interest

None.

## Supplementary Material

supplementary Figure 1A
