# Supplementary material for: Combined Neuropeptide S and D-Cycloserine Augmentation Prevents the Return of Fear in Extinction-Impaired Rodents: Advantage of Dual versus Single Drug Approaches
Source: Int J Neuropsychopharmacol. 2015 Dec 1;19(6):pyv128. doi: 10.1093/ijnp/pyv128 (PMC4926792; doi:10.1093/ijnp/pyv128)
Supplement: supplementary Figure 1A [file ijnp_pyv128_index.html]

Supplementary Data | International Journal of Neuropsychopharmacology

## Supplementary Data

Data files

- Supplementary Data - Supplementary Data
- Supplementary Data - Supplementary Data
