# Supplementary material for: Combined Neuropeptide S and D-Cycloserine Augmentation Prevents the Return of Fear in Extinction-Impaired Rodents: Advantage of Dual versus Single Drug Approaches
Source: Int J Neuropsychopharmacol. 2015 Dec 1;19(6):pyv128. doi: 10.1093/ijnp/pyv128 (PMC4926792; doi:10.1093/ijnp/pyv128)
Supplement: supplementary Figure 1A [file Supplementary_figure_legends.docx]

# Supplementary figure legends

**Figure S1**. Effect of diazepam on fear extinction in HAB and LAB rats. (A) Schematic representation of the experimental design. (B) Freezing levels increased to the same extent in HAB and LAB rats upon five CS-US pairings (pairing effect: F_4,84_ = 65.6, P < 0.001). The systemic application of diazepam (1mg/kg; ip; dissolved in 20 % 2‑hydroxylpropyl‑β‑cyclodextrin; Sigma) 20 min before the extinction training affected the behavior of HAB and LAB rats displayed already before (treatment effect: F_1,22_ = 10.2, P = 0.004) and during the extinction training (CS x treatment interaction: F_14,308_ = 2.33, P = 0.005) and caused enhanced freezing levels in LAB rats on day 3 (line x treatment interaction: F_1,22_ = 4.31, P = 0.049; Supplementary Figure S1) suggesting an extinction-deficit. Data are means ± sem. n = 6‑7 per experimental group. * P < 0.05 for HAB vs. LAB groups, ^a^ P < 0.08 and ^#^ P < 0.05 for drug treatment vs. vehicle treatment. cond: conditioning; DCS: D‑cycloserine; DZP: diazepam; HAB: high anxiety rat; LAB: low anxiety rat.
