# Supplementary figures and images for: Combined Neuropeptide S and D-Cycloserine Augmentation Prevents the Return of Fear in Extinction-Impaired Rodents: Advantage of Dual versus Single Drug Approaches
Source: Int J Neuropsychopharmacol. 2015 Dec 1;19(6):pyv128. doi: 10.1093/ijnp/pyv128 (PMC4926792; doi:10.1093/ijnp/pyv128)

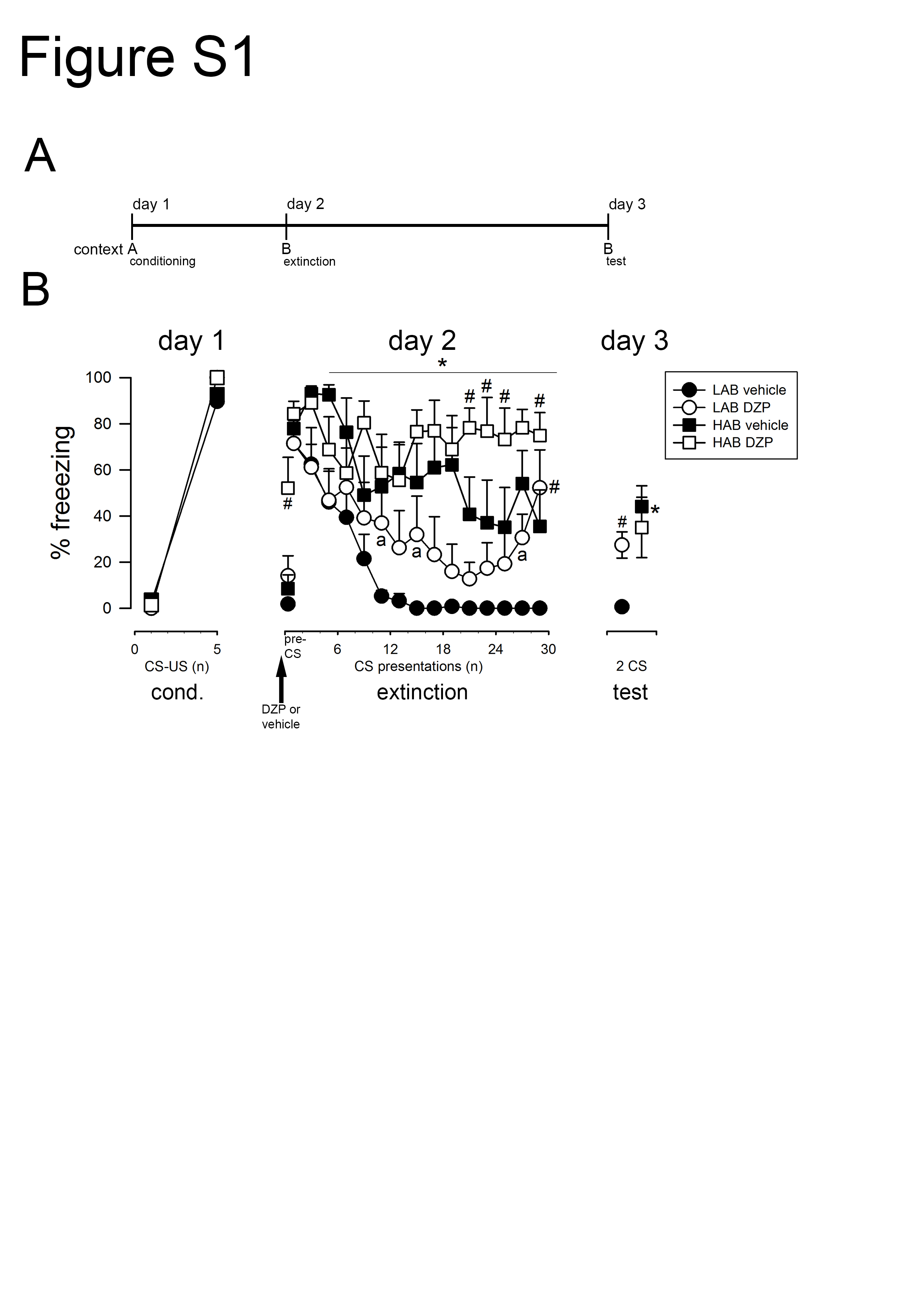

Supplement: supplementary Figure 1A [file S1_NPS_DCS_FigS1_R1.tif]
